# Chemical pulldown combined with mass spectrometry to identify the molecular targets of antimalarials in cell-free lysates

**DOI:** 10.1016/j.xpro.2022.102002

**Published:** 2023-01-06

**Authors:** Robert J. Smith, Rachel Milne, Victoriano Corpas Lopez, Natalie Wiedemar, Gourav Dey, Aisha J. Syed, Stephen Patterson, Susan Wyllie

**Affiliations:** 1 Wellcome Centre for Anti-infectives Research, School of Life Sciences, University of Dundee, Dow Street, Dundee DD1 5EH, UK

**Keywords:** Cell Biology, Cell culture, Microbiology, Molecular/Chemical Probes, Protein Biochemistry, Proteomics, Mass Spectrometry, Chemistry

## Abstract

Here, we provide a protocol using chemical pulldown combined with mass spectrometry (LC-MS/MS) to identify drug targets in *Plasmodium falciparum*. This approach works upon the principle that a resin-bound inhibitor selectively binds its molecular target(s) in cell-free lysates. We describe the preparation of drug beads and *P. falciparum* lysate, followed by chemical pulldown, sample fractionation, and LC-MS/MS analysis. We then detail how to identify specifically bound proteins by comparing protein enrichment in DMSO-treated relative to drug-treated lysates via quantitative proteomics.

For complete details on the use and execution of this protocol, please refer to Milne et al. (2022).^1^

## Highlights

• Unbiased approach to identify the molecular targets of antimalarials
• Preparation of resin-bound probes to bind and identify targets in a cell-free lysate
• Steps to perform chemical pulldown, sample fractionation, and LC-MS/MS analysis
• Protocol can be adapted to study other species

## Abstract (editor highlights)

Publisher’s note: Undertaking any experimental protocol requires adherence to local institutional guidelines for laboratory safety and ethics.

## Graphical abstract

**Figure:**
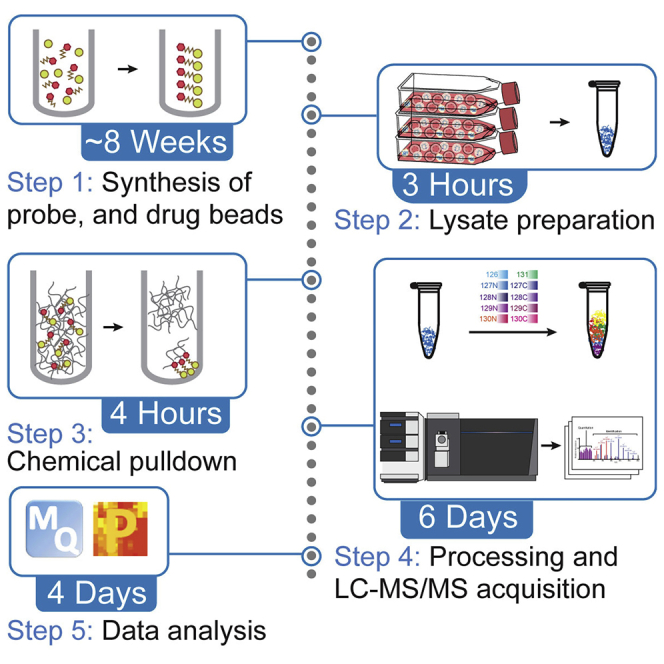

## Before you begin

Understanding the mechanism(s) of action of biologically active compounds at a molecular level can greatly assist the development of drugs and chemical tool compounds. Chemical pulldown is a useful, unbiased technique to identify protein(s) that directly bind to a bioactive molecule of interest. In addition to identifying primary targets linked to a molecule’s mode of action, this approach can also indicate potential off-target liabilities. The successful identification of a protein target by chemical pulldowns is heavily dependent upon both the affinity of the probe for its target(s), and the amount and orientation of probe attached to the drug bead.^2^ This protocol details the steps required to design a probe, control the probe loading level when preparing drug beads, and outlines other quality control measures.

**Figure 1:**
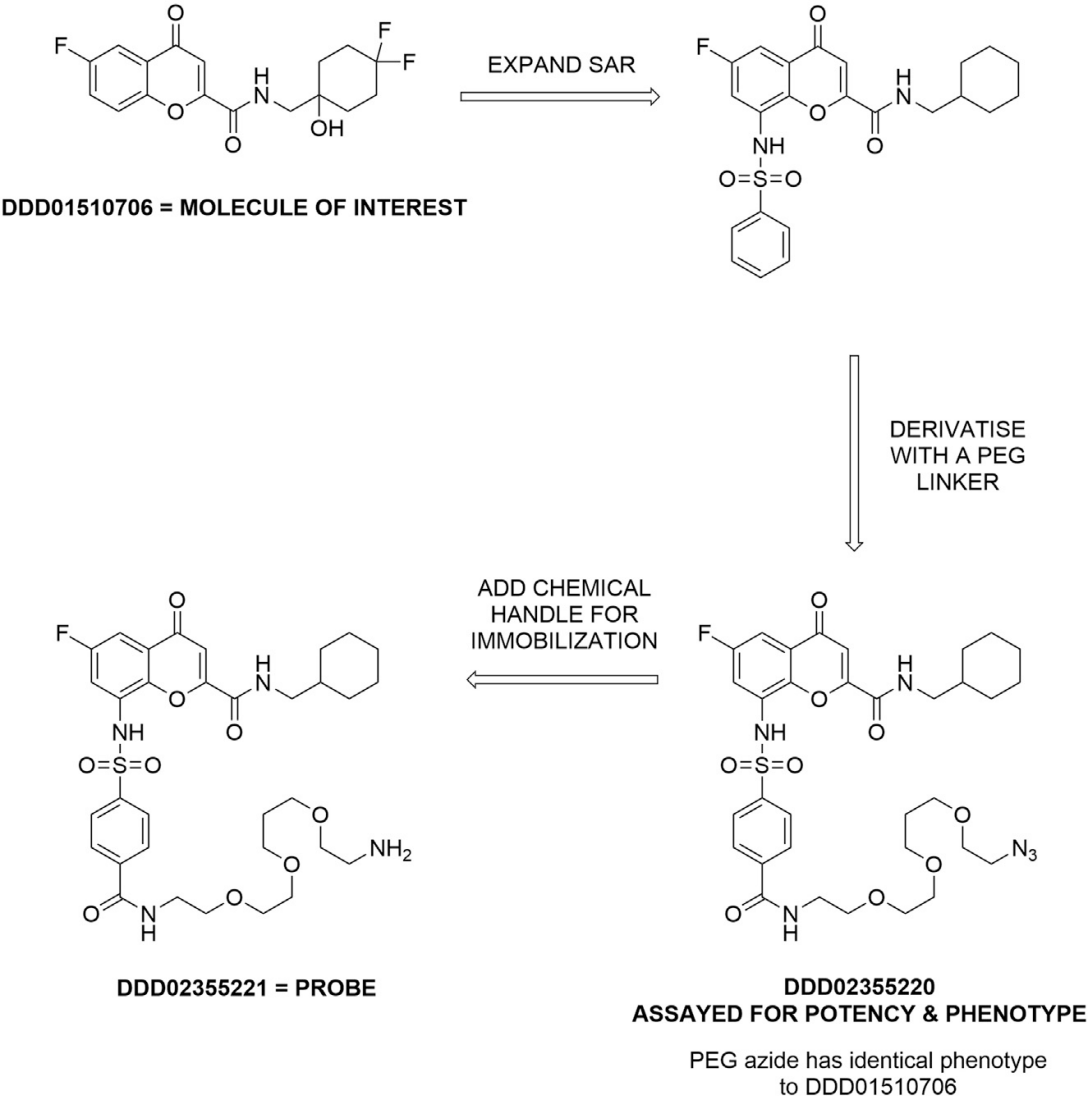
The development of pulldown probe DDD02355221 from the *P. falciparum* KRS inhibitor DDD01510706

The protocol below details the specific steps required to perform pulldown experiments with *Plasmodium falciparum* 3D7 blood stage lysate and Sepharose-based drug beads. We have also used this methodology to identify specific protein-ligand interactions in *Trypanosoma brucei* and *Leishmania donovani*.^3^^,^^4^ The drug bead synthesis protocol specifically details the attachment of **DDD02355221** (Figure 1) onto an activated Sepharose resin.^1^ Probe **DDD02355221** is an analog of **DDD01510706**, a known inhibitor of *P. falciparum* lysyl tRNA synthetase (KRS).^5^ However, we have successfully used this procedure to prepare drug beads based upon other molecules of interest. The specific experiment described herein to exemplify the protocol was designed to identify *P. falciparum* proteins which bind to **DDD01510706**.

This protocol describes the competition variant of a pulldown experiment.^6^ This requires a sample of the bioactive small molecule whose protein binding partners you are aiming to identify. Approximately 25 μL of a 10 mM stock of the molecule of interest is sufficient; the compound should be ≥ 95% pure. Note, additional stock might be required to perform follow up target validation studies. To synthesize a drug bead, it is necessary to prepare, or purchase a small molecule that is suitable for attachment to the resin (see below); the compound should be ≥ 95% pure. Prior to beginning the study prepare 5 L of complete malaria media (CMM; see materials and equipment); this should be pre-warmed to 37°C before use.

### Institutional permissions

*Plasmodium falciparum* is a hazard group 3 human pathogen and must be cultured within a containment level 3 facility. Ensure correct institutional permissions are in place to carry out the work.

### *P. falciparum* asexual blood stage culture

**Timing: approximately 1 week**

1. Culture *P. falciparum* 3D7 (or other suitable strain) in CMM supplemented with 5% A^+^ blood type human erythrocytes.
  ***Note:*** we obtain the A^+^ blood type human erythrocytes from Scottish National Blood Transfusion Service.
  ***Note:*** Incubate the culture at 37°C in a humidified environment of 1% O_2_ and 3% CO_2_ in a balance of N_2_. For routine culture, maintain the parasites in a volume of 10 mL in 25 cm^2^, Thermo Scientific™ Nunc™ EasYFlask™ Cell Culture Flasks with filter cap (or similar).
  a. Change media every 2 days, checking parasitemia by preparing a thin smear and staining using the hemacolor rapid staining of blood smear kit.
    i. Transfer 5 μL of the blood layer to a glass slide and use the edge of a second slide to spread it thinly across the slide. Air dry for approximately 3 min.
    ii. Fix slide in Solution 1 (or 100% methanol) for 1 min then dip (for approximately 0.5 s) 3× in Solution 2, followed by 30 s in Solution 3.
    iii. Rinse off excess stain with H_2_O, dry.
    iv. Count parasitized and non-parasitized erythrocytes using the 100× (oil-immersion) lens of your light microscope and calculate the % parasitemia.^7^
  b. Maintain the culture between 1 and 5% parasitemia, ensuring fresh erythrocytes are provided every 48–72 h.
  c. One week prior to the start of the experiment, begin expanding the culture by increasing the volume in a stepwise manner to a final volume of 150 mL. Use a single 175 cm^2^, Thermo Scientific™ Nunc™ EasYFlask™ Cell Culture Flask with filter cap (or similar).
    **CRITICAL:** If *P. falciparum* grow above 5% parasitemia, more frequent media changes and/or a reduction of the hematocrit are required to maintain the optimal health of the culture. For routine culture do not allow parasites grow above 5% parasitemia.

### Synthesis of compounds for attachment to resin

**Timing: approximately 8 weeks**

The chromone pulldown probe DDD02355221 (Figure 1) was prepared according to the procedure reported in Milne et al. (2022). This required three synthetic steps from starting material prepared under contract by WuXi AppTec. The purity and identity of the DDD02355221 sample used in this study was confirmed by ^1^H-NMR, ^19^F-NMR, ^13^C-NMR and LCMS. DDD02355221 was developed from the reported *Pf*KRS inhibitor DDD01510706 using the general design principles outlined below.^5^

***Note:*** It is necessary to confirm that any pulldown probe retains the bioactivity of the molecule of interest from which it is derived (see below). The time required to profile the probe in the appropriate biological assay(s) (e.g., a phenotypic assay) should be included in the overall study timeline.

***Note:*** The synthetic route towards a compound suitable for attachment onto a resin will be specific for each molecule of interest. As a result, the time to complete probe synthesis will vary significantly depending upon several factors including the complexity of the chemical probe, the availability of robust synthetic routes and knowledge of the structure activity relationship (SAR) of the compound.

### General design principles for pulldown probes

In most cases, phenotypically active molecules are not immediately suitable for attachment onto a resin, and it is necessary to derive a structurally related pulldown probe. The SAR of the compound is used to identify moieties that are likely solvent-exposed and could provide vectors for resin attachment. Using the SAR, analogs functionalized with a polyethyleneglycol (PEG) linker are designed, synthesized and profiled in a phenotypic assay.

**CRITICAL:** If an amine-terminated PEG linker is used, the free amine (e.g., DDD02355221) is highly polar and may have low cell permeability. Therefore, a protected amine (e.g., a Boc carbamate or azide, such as DDD0235520) is used to assess phenotype and potency relative to the parent molecule of interest.

If these PEG-containing analogs retain the biological activity of the parent compound (identical phenotype, ideally within 10-fold potency), they can be progressed as pulldown probes. If there is insufficient SAR data available, a short program to investigate suitable sites of linker attachment may be necessary. The development of probe DDD02355221 required an SAR expansion as outlined in Figure 1. Pulldown probes are attached onto a resin via a covalent bond. Typically, we use *N*-hydroxysuccinimide (NHS)-activated resin, that is functionalized by an amine to form an amide bond.

***Note:*** as chemical pulldown is an affinity-based method the strength of the interaction between the probe and its target(s) affects the outcome of an experiment. During probe development, changes in potency in a phenotypic assay is used as a surrogate for changes in the probe-target binding constant. Therefore, the parent compound of interest should be as potent as possible (ideally low nanomolar) to allow for some loss of potency during probe development. If a molecule of interest is less potent in a phenotypic assay (micromolar range) then a probe with a 10-fold reduction in potency is less likely to result in a successful pulldown. Several molecular properties can influence the potency of a molecule in a phenotypic assay. Therefore, the relationship between a probe’s phenotypic potency and the binding affinity for its target is complex and will be chemotype-dependent.

## Key resources table

**Table:** 

REAGENT or RESOURCE	SOURCE	IDENTIFIER
**Chemicals, peptides, and recombinant proteins**		

Albumax II	Gibco	11021-037
RPMI 1640 (+ 25 mM HEPES, + L-glutamine)	Gibco	13018-031
Sodium bicarbonate	Sigma	S5761
Glucose	Sigma	G7021
Gentamicin (50 mg/mL)	Gibco	15750-037
Hypoxanthine	Sigma	H9377
Sodium hydroxide	Sigma	567530
Hemacolor rapid staining of blood smear	Sigma	1116610001
Dimethyl sulfoxide (DMSO) 99.8+% for molecular biology, DNAse, RNAse and protease free	Acros Organics (via Fisher Scientific)	327182500 (10397841)
N, N-Diisopropyl-ethylamine, 99% (DIPEA)	Fluorochem	005027
Ethanolamine, 99%	Acros Organics (via Fisher Scientific)	149582500 (10033213)
2-propanol, bioreagent for molecular biology >99.5%	Sigma Aldrich	I9516
Cytiva NHS-activated Sepharose 4 fast flow	Cytiva life sciences	17090601
HPLC grade acetonitrile	Fisher Scientific	10660131
HPLC grade water	VWR	23595.328
Formic Acid	Fisher Scientific	10559570
Saponin	PanReac AppliChem	A4518,0100
Potassium acetate	Sigma	P5708
Magnesium acetate	Sigma	M5661
HEPES	Sigma	H4034
Sucrose	VWR	27480.360
Dithiothreitol	Sigma	D0632
Leupeptin hemisulfate	Melford	L22035
cOmplete™, EDTA free protease inhibitor cocktail	Roche	1187350001
Octyl-ß-D-glucopyranoside	Sigma	O8001
TRIS base	VWR	103157P
Ethylenediaminetetraacetic acid tetrasodium salt hydrate	Sigma	E5391
Sodium chloride	VWR	2710.364
Bovine serum albumin (BSA)	BDH	441555J
Quick Start™ Bradford Protein Assay Kit 1	Bio-Rad	5000201
NuPage LDS sample buffer (4×)	Invitrogen	NP0007
NuPage MOPS SDS Running Buffer (20×)	Invitrogen	NP0001
Coomassie quick reagent	NeoBiotech	NB-45-00078
Pierce™ Acetonitrile (ACN), LC-MS grade	Thermo	51101
Formic acid, LC-MS grade	Thermo	85178
Acetonitrile HPLC LC-MS grade	VWR	20J141963
Trifluoroacetic acid HPLC grade	Fisher	T/3258/04
Ammonia solution OPTIMA grade	Fisher	A470-250
Methanol (OPTIMA LC/MS)	Fisher	A456-1
Ammonium formate	Sigma	70221-100G-F
pH-indicator strips pH 0–14 Universal indicator	Sigma	1095350001
Dimethyl sulfoxide, molecular biology grade	Sigma	D8418

**Critical commercial assays**		

TMT10plex™ Isobaric Mass Tagging Kit	Thermo	90111
- Iodoacetamide (IAA)	Thermo (see kit above)	90111
- Triethylammonium bicarbonate (TEAB)	Thermo (see kit above)	90111
- Trypsin	Thermo (see kit above)	90111
- Quenching reagent (50% hydroxylamine)	Thermo (see kit above)	90111
Pierce Peptide Desalting Spin Columns	Thermo	89852

**Deposited data**		

RAW and search files of the project	PRIDE repository, originally reported in Milne et al.^1^	https://www.ebi.ac.uk/pride/archive/projects/PXD033740

**Experimental models: Organisms/strains**		

*Plasmodium falciparum* 3D7 strain, asexual bloodstage parasites	N/A	Taxon ID 36329

**Software and algorithms**		

Thermo XcaliburTM	Thermo	Version 4.0.27.19
ChromeleonTM software	Thermo	N/A
MaxQuant version 1.6.17.0	Tyanova et al.^8^	Maxquant.org
Perseus version 1.6.15	Tyanova et al.^8^	https://maxquant.net/perseus/

**Other**		

NuPAGE 4–12% Bis Tris Gel	Invitrogen	NP0321
Macsmix™ tube rotator	Miltenyi Biotec	130-090-753
Apex 1.5 mL screw-cap microcentrifuge tube	Alpha Laboratories	CP5911
Apex 1.5 mL screw-cap microcentrifuge tube cap	Alpha Laboratories	CP5940W
Glass vial, fixed insert, clear	Agilent Technologies	5188-6591
Glass vial, 2 mL clear	Agilent Technologies	5182-0715
13 mm 0.2 μm syringe filter	Fisher Scientific	15161499
Thermo Scientific™ Nunc™ EasYFlask™ Cell Culture Flasks, 25 cm^2^	Fisher Scientific	156367
Thermo Scientific™ Nunc™ EasYFlask™ Cell Culture Flasks, 175 cm^2^, Filter	Fisher Scientific	159910
Corning™ HYPER*Flask*^TM^ M	Fisher Scientific	10343305
Cell disruption vessel, 45 mL	Parr	4639
Cytiva disposable visible/UV cuvettes	Fisher Scientific	10688095
Protein LoBind® tubes, 0.5 mL	Eppendorf	0030108434
Protein LoBind® tubes, 1.5 mL	Eppendorf	0030108442
Protein LoBind® tubes, 2 mL	Eppendorf	0030108450
Tabletop Optima TLX Ultracentrifuge	Beckman	TLX
Rotor TLA 120.0 Fixed angle rotor	Beckman	362046
Sonicator Elmasonic P60H	Elma	101 3761
Sigma 6–16K centrifuge	C&M Scientific	10395
Vibrax VXR basic shaker	IKA	0002819002
Genevac EZ-2 plus evaporator	SP Scientific	EZ3P-23050-HP0
Dionex UHPLC Ultimate 3000, WPS-3000FC autosampler	Thermo	5825.0020
C18 column XBridge peptide BEH, 130 Å, 3.5 μm, 2.1 × 150 mm	Waters	186003565
Guard column XBridge, C18, 3.5 μm, 2.1 × 10 mm	Waters	186003019
PepMap nanoViper C18 column, 100 μm × 2 cm, 5 μm, 100 Å	Thermo	164564-CMD
Pepswift Monolithic Nano resolving column	Thermo	164584
Thermo Q Exactive Plus Orbitrap System coupled to a Dionex Ultimate 3000 RS	Thermo	IQLAAEGAAPFALGMBDK
Thermo Orbitrap Eclipse System coupled to a Dionex Ultimate 3000 RS	Thermo	FSN04-10000
Thermo Dionex Ultimate 3000 HPLC with diode array detector coupled to an Advion Expression CMS ESI Mass spectrometer connected to an X-Select Column CP C18, 2.5 μM 2.1 × 30 mm	Thermo	IQLAAAGABHFAPBMBEX
Resolving column, 75 μm × 50 cm, PepMap RSLC C18 column, 2 μm, 100 Å	Thermo	164540
Open-top 1 mL thickwall polycarbonate tubes, 11 × 34 mm	Beckman	342778
1.5 mL collection tubes	Sarstedt	72.696

## Materials and equipment

### Suggested equipment and materials

**Choice of mass spectrometer:** This protocol relies on TMT quantitation; therefore pulldown analysis requires an instrument compatible with this technology. We used a Thermo Orbitrap Eclipse instrument, but other machines can be used providing their MS/MS resolution is > 50,000 at 150 m/z and can perform higher-energy collision dissociation (HCD). Suggested equipment includes Orbitrap Velos Pro, Q Exactive HF, Q Exactive Plus, Orbitrap Elite and Orbitrap Fusion. Other TMT products such as TMTsixplex™ or TMTpro™ (16plex or 18plex) can alternatively be used depending on the number of replicates.

**Choice of detergent:** This protocol uses the mild detergent octyl-β-D-glucopyranoside for the cell lysis and pulldown washes. Other non-denaturing detergents can be used as long as they are compatible with mass spectrometry (i.e., they can be removed from the sample before MS analysis). In the past we have successfully used NP-40 as an alternative detergent.

**Table:** Hypoxanthine solution

Reagent	Final concentration	Amount
Hypoxanthine	0.1 M	1.36 g
0.1 M sodium hydroxide	N/A	100 mL
**Total**	**N/A**	**100 mL**

Heat gently to aid dissolution. Store in 10 mL aliquots at −20°C, store for up to 6 months.

**Table:** Complete Malaria Media (CMM)

Reagent	Final concentration	Amount
Albumax II	0.5% (w/v)	25 g
RPMI 1640	N/A	79.45 g
Sodium bicarbonate	12 mM	5 g
Glucose	11 mM	10 g
Gentamicin	20 mg/L	2 mL
Hypoxanthine solution		10 mL
ddH_2_O	N/A	To 5 L
**Total**	**N/A**	**5 L**

Ensure Albumax II has dissolved fully before adding remaining reagents. Adjust pH to 7.3 with sodium hydroxide. Filter sterilize and store at 4°C for up to 2 months. Warm to 37°C before use.

**Table:** Pellet wash buffer

Reagent	Final concentration	Amount
1 M potassium acetate	100 mM	100 mL
1 M magnesium acetate	2.5 mM	2.5 mL
1 M HEPES, pH7.4	45 mM	45 mL
2 M sucrose	250 mM	125 mL
1 M dithiothreitol	2 mM	2 mL
10 mM leupeptin	15 μM	1.5 mL
ddH_2_O	N/A	To 1 L
**Total**	**N/A**	**1 L**

Prepare fresh on day of lysate preparation (dithiothreitol and leupeptin must be added fresh). Use chilled at 4°C.

**Table:** Saponin solution

Reagent	Final concentration	Amount
Saponin	0.1% (w/v)	100 mg
Pellet wash buffer	N/A	100 mL
**Total**	**N/A**	**100 mL**

Prepare fresh on day of lysate preparation. Use chilled at 4°C.

**Table:** Lysis buffer

Reagent	Final concentration	Amount
Pellet wash buffer	N/A	20 mL
Complete protease inhibitor	N/A	1 tablet
**Total**	**N/A**	**20 mL**

Prepare fresh on day of lysate preparation. Use chilled at 4°C.

**Table:** 2× Octyl-β-D-glucopyranoside solution

Reagent	Final concentration	Amount
Lysis buffer	N/A	1 mL
Octyl-β-D-glucopyranoside	1.6% (w/v)	16 mg
**Total**	**N/A**	**1 mL**

Prepare fresh on day of lysate preparation, keep on ice.

**Table:** Pull-down wash buffer

Reagent	Final concentration	Amount
Octyl-β-D-glucopyranoside	0.8% (w/v)	80 mg
1 M Tris-HCl pH8	50 mM	0.5 mL
0.5 M EDTA	5 mM	0.1 mL
BSA	1 mg/mL	10 mg
ddH_2_O	N/A	To 10 mL
**Total**	**N/A**	**10 mL**

Prepare fresh on day of pulldown. Use chilled at 4°C.

**Table:** Tris-buffered saline

Reagent	Final concentration	Amount
1 M Tris-HCl, pH7.5	50 mM	2.5 mL
1 M Sodium chloride	150 mM	7.5 mL
ddH_2_O	N/A	To 50 mL
**Total**	**N/A**	**50 mL**

Store at 4°C for up to 6 months. Use chilled at 4°C.

**Table:** NuPAGE loading buffer

Reagent	Final concentration	Amount
NuPAGE LDS buffer	N/A	250 μL
1 M dithiothreitol	50 mM	50 μL
ddH_2_O	N/A	700 μL
**Total**	**N/A**	**1 mL**

Prepare fresh.

**Table:** 100 mM DIPEA stock solution

Reagent	Final concentration	Amount
DIPEA	100 mM	87 μL
DMSO	N/A	4,913 μL
**Total**	**N/A**	**5 mL**

Store at 4°C, use within 12 weeks.

**CRITICAL:** DIPEA is toxic and flammable, it should be handled and dispensed within a fume cupboard whilst wearing appropriate PPE.

**CRITICAL:** As prepared, the DDD02355221 high and low loading reaction stock solutions have a two-fold excess of DIPEA. If the pulldown probe exists as an acid salt, increase the concentration of this stock solution by 50 mM for each acid equivalent, e.g., a 2HCl salt should use a 200 mM DIPEA stock solution. This is to ensure the reaction maintains a two-fold excess of base relative to the probe compound.

**Table:** 200 mM ethanolamine stock solution

Reagent	Final concentration	Amount
Ethanolamine	200 mM	60 μL
DMSO	N/A	4,940 μL
**Total**	**N/A**	**5 mL**

Store at 4°C, use within 12 weeks.

**CRITICAL:** Ethanolamine is toxic, corrosive and flammable, it should be handled and dispensed within a fume cupboard whilst wearing appropriate PPE.

**Table:** DDD02355221 High loading reaction stock solution

Reagent	Final concentration	Amount
DDD02355221 (50 mM in DMSO)	23 mM	96.6 μL
DIPEA (100 mM in DMSO)	46 mM	96.6 μL
DMSO	N/A	16.8 μL
**Total**	**N/A**	**210 μL**

Prepare immediately before use.

***Note:*** 200 μL of this stock is scaled to be 1 equivalent of probe DDD02355221 and 2 equivalents of DIPEA per reactive NHS group for 200 μL of settled Cytiva Fast Flow 4 Sepharose resin. In our experience this should result in the probe reacting with 60–90% of the available NHS esters on the resin.

**Table:** DDD02355221 Low loading reaction stock solution

Reagent	Final concentration	Amount
DDD02355221 (50 mM in DMSO)	4.6 mM	19.3 μL
DIPEA (100 mM in DMSO)	9.2 mM	19.3 μL
DMSO	N/A	171.4 μL
**Total**	**N/A**	**210 μL**

Prepare immediately before use.

***Note:*** 200 μL of this stock is scaled to be 0.2 equivalents of probe DDD02355221 and 0.4 equivalents of DIPEA per reactive NHS group for 200 μL of settled Cytiva Fast Flow 4 Sepharose resin. In our experience this should result in the probe reacting with 5–15% of the available NHS esters on the resin.

## Step-by-step method details

### Attachment of DDD02355221 to Sepharose resin

**Timing: 3 days**

This section details the attachment of the probe DDD02355221 to Cytiva NHS-activated Fast Flow 4 Sepharose resin at two different loading levels.

**CRITICAL:** Ligand loading levels of drug beads can impact a pulldown experiment.^2^ Our experience to date indicates that there is no way to predict optimal bead loading level for specific probes/target combinations. Thus, our standard approach is to prepare drug beads with two different loading levels, ideally with a >5-fold difference in loading level between the two drug beads. HPLC analysis of the reaction supernatants is used to quantify the amount of probe attached to the drug beads.

**Figure 2:**
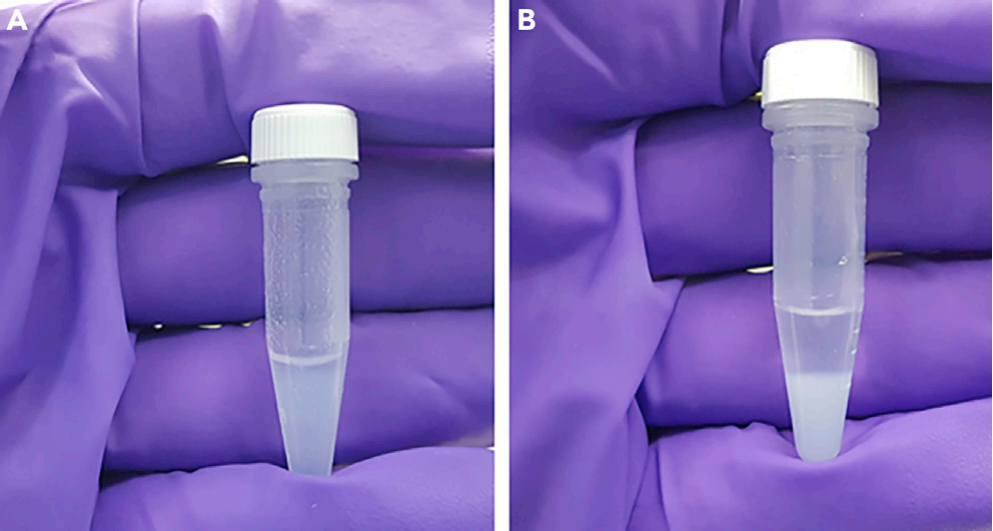
Resin suspended before and after centrifugation (A) NHS-activated Sepharose resin suspended after shaking. (B) pelleted NHS-activated Sepharose resin after centrifugation. Refer to step 1b.

1. Transfer the resin slurry to microcentrifuge tubes.
  a. Add approximately 400 μL of Cytiva NHS activated Sepharose 4 fast flow resin slurry to each of two screw cap microcentrifuge tubes. Label one vial ‘high’ loading level and one ‘low’ loading level.
    ***Note:*** A wide-bore pipette can be used when handling the Sepharose resin for ease of dispensing.
  b. Cap the tube and centrifuge (15,000 g, 30 s, room temperature). Allow the resin to stand for approximately 1 min to settle flat then estimate the volume of settled resin using the volume indicators on the side of the microcentrifuge tube (Figure 2).
    ***Note:*** room temperature is approximately 16°C–20°C.
    ***Note:*** immediately after centrifugation the surface of the resin will be sloped, leaving to stand at room temperature for approximately one minute allows the surface to settle flat (as in Figure 2B) after which supernatant can be removed.
    ***Note:*** high-speed centrifugation can damage some resin types. Low-speed centrifugation at 3–4,000 g will be compatible with most commercial resins.
  c. Add or remove resin as necessary and repeat step 1b so that the volume of settled resin after centrifugation is 200 μL.
    **CRITICAL:** The NHS-activated Fast Flow 4 Sepharose slurry can be difficult to pipette accurately, the quantity of resin used should be determined by the volume indicators on the tube. It is important that the amount of settled resin is up to the 200 μL mark as the amount of NHS groups provided by the manufacturer is quoted per mL of settled resin, not per mL of slurry, and this is used to scale the attachment reaction. Over time (months) the 2-propanol the resin is stored in can slowly evaporate, leading to a thick, unworkable slurry. It may be necessary to periodically add 2–5 mL of molecular biology grade 2-propanol to the slurry to maintain it at a workable viscosity when using the same bottle of resin over an extended period.

2. Wash the resin with DMSO to remove the 2-propanol storage solvent.
  a. Remove the supernatant with a 200 μL pipette, being careful not to disturb the settled resin.
  b. Add 1 mL of molecular biology grade DMSO to the microcentrifuge tube, cap the tube and gently shake to mix.
  c. Centrifuge the tube (15,000 g, 30 s, room temperature) to pellet the resin and carefully remove the supernatant with a 1,000 μL pipette.
  d. Repeat steps 2b and 2c a further two times.

**CRITICAL:** Carefully remove the supernatant to avoid loss of resin, err on the side of caution, it is better to leave excess supernatant than to lose resin during these washing steps. This cautious approach applies to all future washing steps.

**Figure 3:**
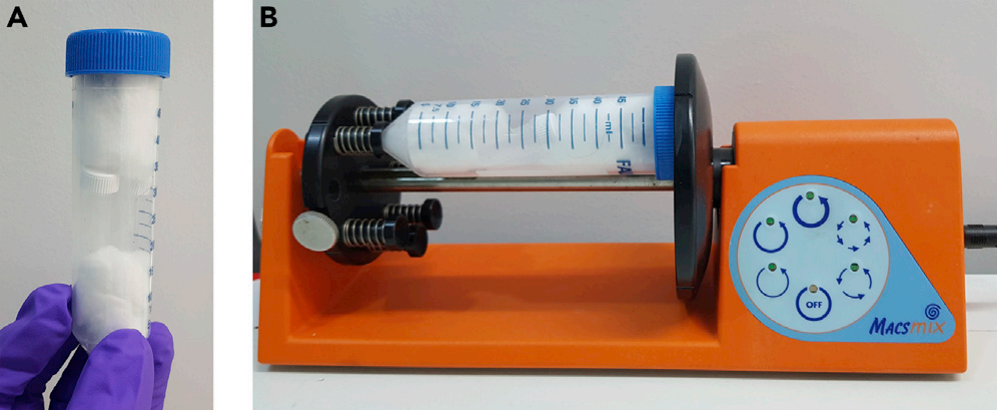
Microcentrifuge vials with resin secured in Falcon^TM^ Tube and rotary mixer setup (A) microcentrifuge tubes secured in 50 mL Falcon tube. (B) Setup of Falcon tube on the MACSmix™ tube rotator.

3. Attachment of DDD02355221 to the Sepharose resin to prepare high- and low-loading drug beads.
  a. After the final washing step and removal of the supernatant, add 200 μL of either the DDD02355221 high loading reaction stock solution, or DDD02355221 low loading reaction stock solution to the appropriately labeled tubes.
  b. Cap the tubes and centrifuge (15,000 g, 30 s, room temperature), then transfer 10 μL of supernatant from each tube to a separate Agilent screw top vial with fixed insert as a t = 0 reading for analysis by HPLC (steps 10 and 11).
  c. Re-cap, then mix both tubes well by inverting several times, then use cotton wool to secure them inside a 50 mL Falcon tube.
  d. Place the Falcon tube on a MACSmix™ tube rotator and rotate at maximum speed setting (20 rpm) for 24 h at room temperature to ensure continued mixing of the resin (Figure 3).

**CRITICAL:** A suitable low volume HPLC vial must be used to allow sampling from 10 μL of supernatant. We use Agilent screw top vials with fixed inserts.

4. Removal of excess DDD023355221.
  a. Following agitation of “high” and “low” drug beads over 24 h, remove tubes from the tube rotator and centrifuge (15,000 g, 30 s, room temperature).
  b. Transfer 10 μL of resulting supernatants from each tube to Agilent screw top vials with fixed insert labeled as t = 24 h “high” and t = 24 h “low”. These samples will be submitted for analysis by HPLC (steps 10 and 11).
  c. Carefully remove the remaining supernatant using a 200 μL pipette.
  d. Add 1 mL of DMSO to each tube, cap the tubes and gently shake to mix.
  e. Centrifuge the tubes (15,000 g, 30 s, room temperature) to pellet the drug beads and carefully remove the supernatant with a 1,000 μL pipette.
  f. Repeat steps 4d and 4e a further two times.

***Note:*** If the probe being attached is highly colored, the drug beads may be stained that color after all washing steps are complete.

5. Block the drug beads with ethanolamine to quench unreacted NHS-ester groups.
  a. Add 200 μL of 200 mM ethanolamine stock solution to each tube, cap, invert and shake the tubes gently to mix the drug beads.
  b. Secure the microcentrifuge tubes using cotton wool inside a 50 mL Falcon tube as previously described (see step 3c). Place on a MACSmix™ tube rotator and rotate at maximum speed setting (20 rpm) at room temperature for 24 h to ensure continued mixing of the drug beads.
  c. After 24 h pellet the drug beads by centrifugation (15,000 g, 30 s, room temperature) and carefully remove the supernatant from each tube with a 200 μL pipette.
  d. Add 1 mL of DMSO to both tubes, cap and gently shake to mix the drug beads.
  e. Centrifuge the tubes (15,000 g, 30 s, room temperature) to pellet the drug beads and carefully remove the supernatant with a 1,000 μL pipette.
  f. Repeat steps 5d and 5e a further two times.
6. Transfer drug beads to storage solution.
  a. Add 1 mL of 2-propanol to both tubes, invert and shake gently to mix the drug beads.
  b. Centrifuge (15,000 g, 30 s, room temperature) and remove the supernatant from each tube with a 1,000 μL pipette.
  c. Repeat steps 6a and 6b a further two times.
  d. Estimate the volume of settled drug beads using the volume indicators on the side of the microcentrifuge tubes and add 2-propanol to make a 1:1 slurry.

***Note:*** Assuming minimal loss of resin, approximately 200 μL of 2-propanol should be added to each tube in step 6d.

**CRITICAL:** It is important that as close as possible to a 1:1 drug bead:2-propanol slurry is prepared, as this is used to standardize the amount of resin used in subsequent pulldown experiments.

**CRITICAL:** Do not progress the drug beads into a pulldown experiment until analysis of probe attachment (steps 7–11) has been completed.

**Pause point:** At this stage the drug beads can be stored at 4°C until needed.

***Note:*** There is no straightforward method to assess the condition of the ligand on the drug bead after attachment. Therefore, it is preferable to perform pulldown experiments as close to the time of synthesis as practically possible. Although we have successfully repeated pulldown experiments on a batch of resin that was several months old, we cannot guarantee that this is broadly applicable.

### Establishing efficiency of probe attachment to the resin

**Timing: 1 day**

***Note:*** This is section of the protocol requires 1–2 h for sample preparation, 5–10 h for HPLC analysis then approximately 1 h for data processing.

This section details the HPLC analysis of the reaction supernatant collected at t = 0 and t = 24 h for both high- and low-loading drug beads using a Thermo Dionex Ultimate 3000 HPLC with diode array detector coupled to an Advion Expression CMS ESI Mass spectrometer connected to an X-Select Column CP C18, 2.5 μM 2.1 × 30 mm. This method assumes that any probe compound removed from the reaction solution has attached to the resin. Therefore, measuring the amount of probe remaining after 24 h allows the loading level to be estimated.

7. Prepare stock solutions for an eight-point standard curve by serial dilution from a 50 mM DMSO stock of DDD02355221; volumes used at each dilution step are outlined in the following table:

**Table:** 

[DDD02355221] (mM)	Volumes used for serial dilution starting with 50 mM DDD02355221 (μL)	DMSO (μL)
50	–	–
23	46	54
15	65.2	34.8
10	66.7	33.3
5	50	50
2.5	50	50
1.25	50	50
0.625	50	50
0.3125	50	50

8. Prepare standard curve and quality control samples for HPLC. Troubleshooting 1.
  a. Pipette 10 μL of each standard curve stock solution (excluding 50 mM) into a low volume HPLC vial.
  b. Dilute with 190 μL of 1:1 MeCN/water and mix.
  c. Independently prepare 10 mM, 1 mM and 0.1 mM stock solutions of DDD02355221, to serve as quality control samples to validate the accuracy of the standard curve.
  d. Pipette 10 μL of each validation stock solution into a low volume HPLC vial.
  e. Dilute with 190 μL of 1:1 MeCN/water and mix.

***Note:*** The quality control samples prepared in step 8c should be made from a fresh 10 mM probe stock solution. However, if there is insufficient probe compound to prepare these quality control samples, they can be prepared by dilution of the 50 mM probe stock solution used in step 7.

9. Dilute the t = 0 h and t = 24 h aliquots for the high- and low-loading drug beads collected in steps 3b and 4b respectively with 190 μL of 1:1 MeCN/water, mix and filter through a 13 mm 0.2 μm syringe filter.
10. Analyze the standard curve, quality control and experimental samples in triplicate using HPLC.
  a. Water with 0.1% (v/v) formic acid and acetonitrile were used as the A and B HPLC solvents respectively.
  b. Set up a method with a flow rate of 0.800 mL min^-1^ and injection volume of 3 μL. Perform a 30 s pre-equilibration at 95:5 (A:B), then a linear gradient of 95:5 → 5:95 (A:B) from 0.0 to 2.2 min, with a hold at 5:95 (A:B) from 2.2 to 3.0 min, then 95:5 (A:B) from 3.0 to 4.0 min.
  c. Analyze samples from steps 8b, 8e and 9 in triplicate using the method from 10b.

**CRITICAL:** use HPLC and MS conditions appropriate for the probe that is being attached to the drug bead.

***Note:*** we use a Thermo Dionex Ultimate 3000 HPLC connected to a C18 reverse phase column. However, any HPLC setup suitable for chemical reaction monitoring will suffice.

11. Construct a DDD02355221 standard curve using the UV chromatogram peak areas. From this calculate the concentration of DDD02355221 in the samples at t = 0 h and t = 24 h.
  ***Note:*** These calculations are readily performed using a spreadsheet (Data S1). A copy of the Microsoft Excel file used in our laboratory is provided as supporting information. Troubleshooting 2.
  a. Input the peak areas for peaks corresponding to the probe in the UV chromatograms from all samples into the corresponding cells in the provided excel spreadsheet (Data S1).
  b. Fill in the aX + bY values from the standard curve equation into the corresponding cells.
  c. The rest of the excel spreadsheet will automatically populate, providing the concentration of samples, the amount of DDD02355221 remaining in solution, the amount of DDD02355221 attached to the resin and the drug bead percentage loading level (the percentage of NHS esters that have reacted).

**CRITICAL:** The concentration of the 10 mM, 1 mM and 0.1 mM quality control samples (prepared in step 8e) should be determined from the standard curve. A discrepancy of >25% between the calculated and expected concentration of two of the three quality controls suggests an error. The discrepancy could result from an error in the dilutions to make the standard curve, or in the preparation of the quality control samples. If this is the case, the data provided in the following steps may not be accurate.

**CRITICAL:** This HPLC analysis is a vital drug bead quality control procedure, if there is no decrease in the concentration of probe between the t = 0 h and t = 24 h samples, then the probe has not attached to the resin and the drug beads should not be used in a pulldown experiment (see troubleshooting 2). Ideally drug beads used for pulldown experiments should have >5% loading.

***Note:*** Once the t = 0 h and t = 24 h peak areas have been entered into the spreadsheet a qualitative assessment of resin loading is calculated. This is based upon the ratio of the peak areas at t = 24 h and t = 0 h. In our experience this provides an estimation of the probe loading level within 1–5 percent of the value calculated using the standard curve.

### Preparation of blank resin

**Timing: 2 days**

***Note:*** The preparation of blank beads requires approximately 45 min of preparation time on day 1. The bead-forming reaction takes 24 h to reach completion, followed by approximately 45 min of further laboratory time on day 2. This part of the protocol can be performed in parallel with steps 5 and 6 above.

This section details preparation of blank resin that is used in the pulldown protocol described below. In a blank resin all the NHS esters are reacted with ethanolamine.

12. Transfer resin slurry to a microcentrifuge tube, wash and resuspend in DMSO.
  a. Add 400 μL of Cytiva NHS-activated Sepharose 4 Fast Flow resin slurry to a 1.5 mL screw cap microcentrifuge tube.
  b. Cap the tube, centrifuge (15,000 g, 30 s, room temperature) then allow to stand for approximately 1 min to settle.
  c. Carefully remove the supernatant with a 200 μL pipette, being careful not to disturb the settled resin.
  d. Add 1 mL of DMSO to the tube, cap, invert and gently shake to mix.
  e. Centrifuge (15,000 g, 30 s, room temperature) to pellet the resin, then remove the supernatant carefully with a 1,000 μL pipette.
  f. Repeat steps 12d and 12e two further times.
13. Resin blocking reaction.
  a. Add 200 μL of 200 mM ethanolamine stock solution to the settled resin, cap the microcentrifuge tube, invert and shake gently to mix.
  b. Secure the microcentrifuge tube using cotton wool inside a 50 mL Falcon tube and place on a MACSmix™ tube rotator at maximum rotation (20 rpm) for 24 h at room temperature to ensure continued mixing of the resin.
  c. Pellet the resin by centrifugation (15,000 g, 30 s, room temperature) and the carefully remove the supernatant with a 200 μL pipette.
  d. Add 1 mL of DMSO to the pelleted resin, cap the tube and gently shake to mix.
  e. Centrifuge (15,000 g, 30 s, room temperature) to pellet the resin and carefully remove the supernatant with a 1,000 μL pipette.
  f. Repeat steps 13d and 13e a further two times.
14. Transfer resin to storage solution.
  a. Add 1 mL of 2-propanol to the blank resin, cap the tube, invert and shake gently to mix.
  b. Centrifuge (15,000 g, 30 s, room temperature) and remove the supernatant with a 1,000 μL pipette.
  c. Repeat steps 14a and 15b a further two times.
  d. Estimate the volume of settled resin using the volume indicators on the side of the microcentrifuge tube and add 2-propanol to make a 1:1 slurry.

***Note:*** Assuming minimal loss of resin, approximately 200 μL of 2-propanol should be added to the resin in step 14d.

**Pause point:** At this stage the blank resin can be stored at 4°C until needed. Blank resin can be prepared up to 4 weeks in advance of performing a pulldown experiment.

### Culture of asexual blood stage *P. falciparum*

**Timing: 3–5 days**

Each pulldown experiment (consisting of two separate pulldowns, one with low- and one with high-loading drug beads) requires approximately 5 mg total protein; a 500 mL *P. falciparum* culture should provide sufficient protein for the experiment. Scale-up as necessary for more complex studies.

15. Around 3 days before starting the pulldown experiment, decrease the hematocrit to 2% and transfer a total volume of approximately 560 mL of culture to a HYPER*Flask*^TM^.
  a. Use a 150 mL culture at 5% hematocrit with parasitemia around 4–5%. Increase the volume to 375 mL by addition of CMM to reduce the hematocrit to 2%. Then add 150 mL of CMM/2% erythrocytes to obtain a volume of 525 mL and carefully pour into the HYPER*Flask*^TM^:
    i. Angle the HYPER*Flask*^TM^ away from you and pour the culture in a steady stream being careful to avoid creating bubbles. Top up with more CMM/2% erythrocytes ensuring the flask is completely full and no free space remains in the HYPER*Flask*^TM^ (total volume approximately 560 mL).
    ii. With the HYPER*Flask*^TM^ now upright, tap the sides firmly by hand to release any air bubbles before securing the lid.
    iii. Invert the HYPER*Flask*^TM^ several times to mix, then incubate (37°C, 1% O_2_ and 3% CO_2_ in a balance of N_2_).
16. Refresh media 1 or 2 times per day (see CRITICAL note below) and check parasitemia daily by preparing a thin smear (see before you begin, step 1a).
  a. Around 1 h before changing the media, stand the HYPER*Flask*^TM^ upright to allow the blood to settle.
  b. With the HYPER*Flask*^TM^ at an angle, aspirate off approximately one third of the culture volume without disturbing the erythrocytes.
  c. Refill the HYPER*Flask*^TM^ with CMM (supplemented with 0.4% erythrocytes every second day), release air bubbles and mix culture well as outlined in step 15.
17. Proceed with the preparation of the lysate when parasitemia reaches 8–12% (steps 18–27).

***Alternatives:*** If preferred, the HYPER*Flask*^TM^ can be replaced with multiple 175 cm^2^ or 225 cm^2^ cell culture flasks. Keep the hematocrit at 2–2.5% and change media once to twice a day to allow for a high parasitemia and to keep the culture healthy.

**CRITICAL:** If cultures reach a high parasitemia without sufficient media changes, the parasite viability quickly decreases. Therefore, refresh the media at the beginning and end of each day as parasitemia rises above 5%.

### Preparation of *P. falciparum* lysate

**Timing: 3 h**

Preparation of the *P. falciparum* lysate for pulldown analysis requires erythrocyte lysis and removal of erythrocyte material followed by lysis of the parasites. Before you begin, pre-chill the pellet wash buffer, saponin solution, lysis buffer and Octyl-β-D-glucopyranoside solution on ice. Also, rinse the cell disruption vessel chamber with lysis buffer and pre-chill on ice prior to preparing the lysate.

18. Stand up the HYPER*Flask*^TM^ to allow erythrocytes to settle for 1 h. Aspirate off about 20% of the media from the top and transfer culture to centrifuge tubes.
19. Pellet cells by centrifugation (1,800 g, deceleration setting 2, room temperature, 15 min).
20. Set centrifuge to chill to 4°C.
21. Resuspend pelleted cells in 5 pellet volumes of ice-cold saponin solution and incubate on ice for 10 min, with vigorous mixing every 2–3 min to lyse the erythrocytes.
22. Centrifuge the resuspended cells (2,800 g, deceleration setting 5, 4°C, 8 min).
23. Carefully aspirate off the supernatant and wash the free parasites 3 times with pellet wash buffer.
  ***Note:*** After saponin treatment, the pellet will be almost black and the supernatant a very dark reddish-brown color. It can be difficult to distinguish between them, so take extra care when aspirating.
  a. Aspirate supernatant and resuspend pellet in 25 mL pellet wash buffer.
  b. Centrifuge the resuspended pellet (2,800 g, deceleration setting 5, 4°C, 8 min).
  c. Repeat twice more. Supernatant should be a pale straw-colored solution after the final wash. If the supernatant is still pink, perform an additional wash step. Troubleshooting 3.
24. Following the final wash, resuspend the pellet in 0.5 pellet volumes of lysis buffer, mix well ensuring no clumps remain and transfer directly to the chamber of the cell disruption vessel (Figure 4).

**Figure 4:**
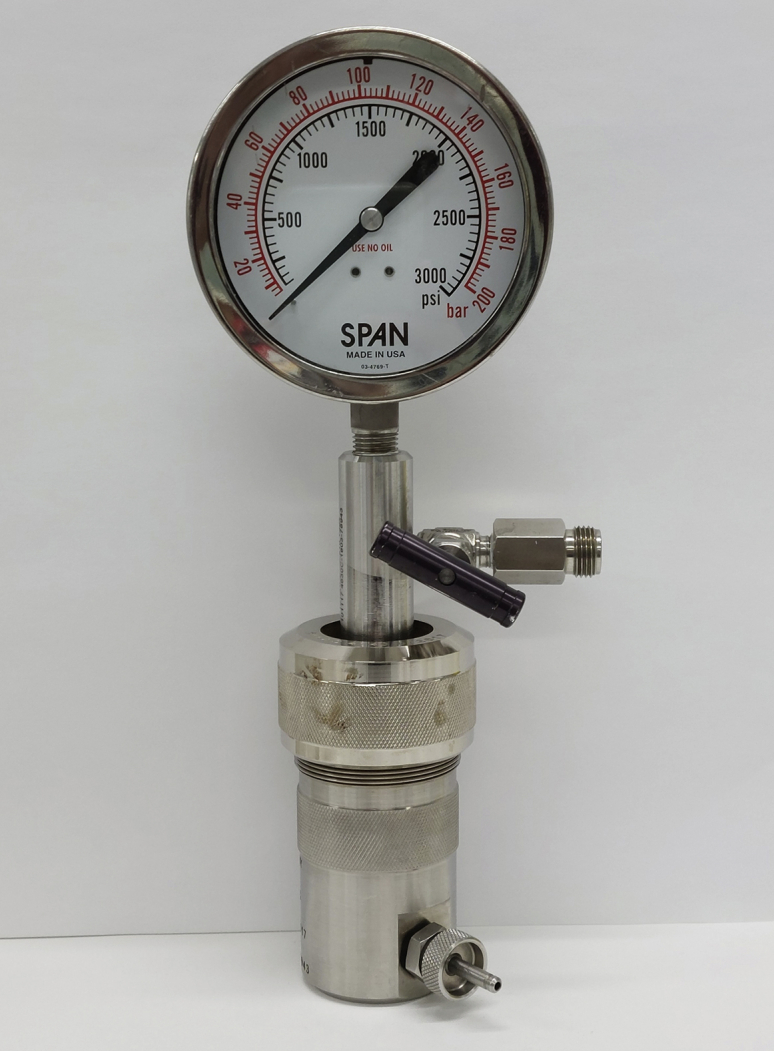
Cell disruption vessel Parasites are ruptured by pressure in the cell disruption vessel at 1,500 psi. (we use a 45 mL vessel from Parr).
25. Add 0.5 pellet volumes of octyl-β-D-glucopyranoside solution and close the chamber tightly.
26. Connect the cell disruption vessel to a nitrogen cylinder and adjust pressure to 1,500 psi. Incubate on ice for 60 min.
27. Very carefully open the pressure valve, releasing the lysate dropwise into a 50 mL falcon tube.

**CRITICAL:** Do not release the pressure too quickly, as the lysate could spray out.

### Pulldown experiment

**Timing: 4 h**

The parasite lysate is first incubated with blank beads prior to incubation with an excess of competitor (parent compound) or DMSO. Drug beads are then added to the pre-treated lysates. Following incubation, beads are then washed, loaded on a Bis-Tris polyacrylamide gel and bead-bound protein electrophoresed a short way into the gel. Protein loaded onto the gel is visualized using a protein stain, the entire stained protein band is then excised from the gel prior to further processing.

28. Centrifuge the lysate (20,000 g, 4°C, 15 min).
29. While lysate is being centrifuged, wash the blank, “high” and “low” loaded drug beads.
  a. Vortex the bead slurry, then transfer 20 μL/sample to a LoBind® Eppendorf tube.
  b. Add 1 mL ddH_2_O to each resin sample.
  c. Centrifuge the resin samples (10,000 g, 30 s, room temperature).
  d. Carefully remove the supernatants with a pipette and discard.
  e. Repeat steps 29b–29d.
  f. repeat steps 29b–29d twice more substituting the ddH_2_O with lysis buffer (containing 0.8 mg/mL Octyl-β-D-glucopyranoside).
30. Transfer lysate supernatant to a fresh LoBind® tube and keep on ice.
31. Measure protein content of lysate using Bio-Rad Quick Start™ Bradford kit.
  a. Prepare a BSA standard curve by diluting the provided 2 mg/mL BSA solution to 0.5 mg/mL in ddH_2_O, then adding the following volumes to each of six 70 μL 10 mm path length UV-visible spectrophotometer cuvettes: 0, 1.25, 2.5, 5, 10, 20 μL.
  b. Add 0.5 μL and 1 μL of lysate respectively to two cuvettes.
  c. Add 1 mL Bradford reagent to each cuvette and incubate at room temperature for 5 min.
  d. Measure the absorbance at 595 nm using a spectrophotometer.
  e. Use the standard curve based on the following table to estimate lysate protein concentration.

**Table:** 

Volume BSA (μL)	Protein concentration (mg/mL)
0	0
1.25	0.625
2.5	1.25
5	2.5
10	5
20	10

***Alternatives:*** Other protein quantitation methods can be used (bicinchoninic acid or BCA, and UV) taking into account contaminants in the lysis buffer such as detergents.

32. Dilute lysate to 2 mg/mL in lysis buffer. You will need 2 mg of protein per drug bead to complete a pulldown (1 mg each for +/- competitor incubations).
33. Incubate lysate with pre-washed blank beads (from step 29), rotate on a MACSMix™ rotator (maximum setting; 20 rpm) in a 4°C cold room for 30 min.
34. Centrifuge (10,000 g for 30 s, room temperature), remove the supernatant and split between two fresh LoBind® tubes (500 μL supernatant each). Add DMSO (5 μL) to one tube, and 10 mM DDD01510706 DMSO stock (5 μL; 0.1 mM final concentration) to the second tube.

***Note:*** A high concentration, or vast excess, of competing ligand is desirable to maximize the shift in enrichment between control and drug-treated samples. In this case 0.1 mM was chosen to limit the final concentration of DMSO to 1%.

**CRITICAL:** if the aqueous solubility of the competitor compound is < 0.1 mM reduce the amount of 10 mM stock solution added to the blank bead-treated lysate in step 34.

35. Incubate for 30 min at 4°C with rotation (as for step 33).
36. Transfer the blank bead-treated lysate samples to tubes containing the pre-washed drug beads (from step 29) and incubate at 4°C with rotation (as for step 33) for 1 h.
37. Centrifuge (10,000 g, 30 s, room temperature), then discard the supernatant and wash 2× with 0.5 mL ice-cold wash buffer, then 2× with 0.5 mL ice-cold TBS.
38. After final wash, add 25 μL NuPAGE buffer and heat at 95°C in a heat block for 10 min.
39. Remove the samples from the heat block and incubate at room temperature for 5 min before loading onto a 4–12% NuPAGE Bis-Tris gel leaving 2 empty lanes between each sample to prevent cross over.
40. Electrophorese the gel at 150 V for 10 min in NuPAGE 1×MOPS SDS Running Buffer.
41. Remove the gel from its cassette and then submerge it in Coomassie Quick Reagent with gentle agitation using a benchtop orbital shaker or equivalent for 60 min. Now aspirate the Coomassie reagent and gently rinse the gel with ddH_2_O.
42. Use a scalpel blade to cut out each lane and transfer the gel slices to labeled LoBind® tubes.

**Pause point:** Samples can now be stored at −20°C for a maximum of 3 weeks prior to further processing.

### Sample processing, digestion and TMT labeling

**Timing: 3 days**

This section describes the process of reducing, alkylating and digesting proteins in the excised gel slice. In addition, the process of labeling peptides with TMT 10-plex tags and then desalting are detailed.

**CRITICAL:** Trifluoroacetic acid (TFA) and formic acid (FA) are harmful when inhaled. They need to be used in a fume hood to minimize the risk of inhalation and disposed following the appropriate health and safety regulations. Wear appropriate PPE when handling TFA or FA.

43. Transfer the gel band into a 1.5 mL tube using clean tweezers and cut the gel into small pieces using a scalpel.
44. Wash the gel pieces with 200 μL water (milliQ) at room temperature with agitation (600 rpm) for 15 min, centrifuge (3,000 g, 1 min) and remove washing solution.

***Note:*** sample agitation is performed using a Vibrax VXR basic shaker.

45. Wash the gel pieces with 200 μL acetonitrile at room temperature with agitation (600 rpm) for 15 min to dehydrate the gel pieces, centrifuge (3,000 g, 1 min) and remove washing solution.
46. Wash the gel pieces with 200 μL 100 mM tetraethylammonium bicarbonate pH 8.5 (TEAB) at room temperature with agitation (600 rpm) for 15 min, centrifuge (3,000 g, 1 min) and remove washing solution.
47. Wash the gel pieces with 200 μL 50% acetonitrile in 100 mM TEAB at room temperature with agitation (600 rpm) for 15 min, centrifuge (3,000 g, 1 min) and remove washing solution.
48. Wash the gel pieces with 100 μL acetonitrile at room temperature with agitation (600 rpm) for 10 min, centrifuge (3,000 g, 1 min) and remove washing solution.
49. Vacuum dry the samples in an evaporator for 10 min at 30°C using a program suitable for water-based samples (program “Aqueous” in the recommended evaporator).
50. Add 50 μL (or enough volume to cover the gel, whichever is greater) 10 mM dithiothreitol in 100 mM TEAB and incubate for 60 min at 56°C, centrifuge (3,000 g, 1 min) then remove all the liquid. Let the samples equilibrate to room temperature (22°C) for 5 min.
51. Add 50 μL (or enough volume to cover the gel, whichever is greater) 50 mM iodoacetamide in 100 mM TEAB and incubate for 30 min at room temperature, centrifuge (3,000 g, 1 min) then remove all the liquid.

**CRITICAL:** Use a fresh IAA solution and preserve it from light. Foil can be used to protect alkylated samples and IAA solution from light. IAA is a sensitizer and samples should be prepared and handled in a fume cupboard whilst wearing appropriate PPE.

52. Wash the gel pieces with 200 μL 100 mM TEAB at room temperature with agitation (600 rpm) for 15 min, centrifuge (3,000 g, 1 min) and remove washing solution.
53. Wash the gel pieces with 200 μL 50% acetonitrile in 100 mM TEAB at room temperature with agitation (600 rpm) for 15 min, centrifuge (3,000 g, 1 min) and remove washing solution.
54. Wash the gel pieces with 100 μL acetonitrile at room temperature with agitation (600 rpm) for 10 min, centrifuge (3,000 g, 1 min) and remove washing solution.
55. Vacuum dry the samples in an evaporator for 10 min at 30°C using a program suitable for water-based samples (program “Aqueous” in the recommended evaporator). Gel pieces will have reduced to ¼ of their initial size.
56. Add 10 μL 12.5 mg/mL trypsin (or enough volume to cover the gel pieces, for approximately 25:1 protein:enzyme ratio) in 100 mM TEAB and incubate for 30 min at room temperature with agitation (600 rpm) to allow rehydration of the gel pieces.

***Note:*** If required, add extra 100 mM TEAB solution to cover the gel pieces.

57. Incubate samples for >16 h at 30°C with agitation (600 rpm).
58. Add 1 volume of acetonitrile and incubate at 30°C for 15 min with agitation (600 rpm). Transfer the liquid from this tube (tube 1) to a new tube (tube 2); this contains the peptides.
59. Add 1 volume of 5% (v/v) aqueous formic acid (FA) to tube 1 with the gel and incubate at 30°C with agitation (600 rpm) for 15 min.
60. Add 1 volume of acetonitrile to tube 1 with the gel pieces and incubate at 30°C with agitation (600 rpm) for 45 min. Then transfer all liquid to tube 2 containing the peptides.
61. Vacuum dry tube 1 in an evaporator at 30°C using a program suitable for water-based samples (program “Aqueous” in the recommended evaporator).
62. Add 10 μL acetonitrile to the gel pieces in tube 1 and incubate at 30°C for 15 min with agitation (600 rpm). Transfer all liquid to tube 2. Tube 1 can now be discarded.
63. Vacuum dry the peptide samples in an evaporator overnight (8–16 h) at 30°C using a program suitable for water-based samples (program “Aqueous” in the recommended evaporator).

**Pause point:** Peptides can be stored at −20°C for a maximum of 6 months.

***Optional:*** We recommend checking the digestion efficiency of some of the samples via mass spectrometry prior to TMT labeling.

64. To check the digestion efficiency:
  a. Resuspend these samples in 100 μL 100 mM TEAB solution, then pick a 1 μL aliquot and dilute it 1:5 in 0.1% (v/v) aqueous FA in a sample tube.
  b. Use the following recommended equipment: Q Exactive Plus mass spectrometer coupled to a Dionex Ultimate 3000 RS (Thermo Scientific) equipped with a trap column (100 μm × 2 cm, PepMap nanoViper C18 column, 5 μm, 100 Å, Thermo Scientific) and a Pepswift Monolithic Nano resolving column.
  c. Place each sample in the sample rack of the HPLC and load 5 μL sample at 10 μL min^-1^ onto the trap column pre-equilibrated with Buffer C.
  d. Wash the trap column with Buffer C for 3 min at 10 μL min^-1^, then switch the trap column in line with the resolving column.
  e. Elute peptides at a constant flow rate of 700 nL min^−1^ with a linear gradient from 2%–40% Buffer D over 7 min, and then from 40%–98% Buffer D over 1 min.
  f. Wash the column with 98% Buffer D for 1 min and re-equilibrate in 2% Buffer D for 5 min.
  g. Use Q Exactive Plus in data-dependent positive ion mode using the following setup:
    i. MS1 scan cycles m/z range from 335–1,800, with a maximum ion injection time of 30 ms, a resolution of 70,000 and automatic gain control (AGC) value of 1 × 10^6^.
    ii. Followed by 10 sequential dependent MS2 scans with an isolation window set to 1.4 m/z, resolution at 17,500, maximum ion injection time at 100 ms and AGC 2 × 10^5^.
  h. Open the .raw files using Thermo Xcalibur™ (Qual browser). This should open two windows: the upper window showing a chromatogram of signal relative abundance over retention time and the lower window showing the relative abundance over m/z ratio.
  i. By selecting peaks on the upper chromatogram, the lower window will display the relative abundance and m/z of that peak.

***Note:*** Check that most peaks have a charge lower than 4 (z < 4). A high abundance of multiply charged species indicates that digestion has been not accomplished and the digestion step should be repeated with fresh digestion enzyme.

65. TMT 10plex labels are provided in sachets stored at −20°C. Remove 1×TMT 10plex sachet from storage at −20°C and allow to equilibrate to room temperature.

***Note:*** Each sachet contains 10 tubes with 0.8 mg of the corresponding TMT 10plex label, enough to label 100 μg of protein.

***Note:*** Unused TMT reagents can be stored back at −20°C in sealed bags in the presence of desiccant.

66. Resuspend the dried peptide samples in 100 mM TEAB (100 μL) and incubate at room temperature with agitation (600 rpm).

***Note:*** Check that the pH of samples is ≥ 8.

67. Add 41 μL of acetonitrile to each TMT tube and incubate with agitation (600 rpm) for 5 min at room temperature.
68. Briefly, centrifuge peptide samples and TMT tubes (3,000 g, 10 s).
69. Add the content of each TMT tube to the corresponding peptide sample.
70. Incubate with agitation for 1 h at room temperature.
71. Dilute the quenching reagent (50% hydroxylamine solution) 10-fold in 100 mM TEAB, making a final 5% hydroxylamine solution.

***Note:*** If the quenching reagent has precipitated, incubate at 37°C until the solution clears.

72. To quench the residual, unreacted TMTs, add 5% hydroxylamine solution (8 μL) to each reaction tube and incubate with agitation at room temperature for 15 min.
73. Centrifuge (3,000 g, 10 s, room temperature) each reaction tube and then combine the 10× treated samples together in a single LoBind® 2 mL tube. Then combine the 10× control samples into another 2 mL LoBind® tube.
74. Vacuum dry the pooled samples in an evaporator overnight (8–16 h) at 30°C using a program suitable for water and organic solvent-based samples (program “HPLC” in the recommended evaporator).

**Pause point:** TMT-labeled peptides can be stored at −20°C for a maximum of 6 months.

75. To start the desalting step, resuspend the dried pooled samples in 0.1% (v/v) aqueous TFA (300 μL). Desalting is carried out with the Pierce Desalting Columns in a centrifuge at room temperature.
76. Place the desalting columns (one per sample) in 2 mL LoBind® tubes and centrifuge (5,000 g, 1 min) to remove the equilibration buffer. Discard the flow through.
77. Wash the desalting columns 2× with 100% ACN (300 μL, 5,000 g, 1 min, room temperature) and discard the flow-through.
78. Wash the columns a further 2× with 0.1% (v/v) aqueous TFA (300 μL, 5,000 g, 1 min, room temperature) and discard the flow-through.
79. Add the samples to separate desalting columns, centrifuge (3,000 g, 1 min, room temperature) and discard the flow-through. The peptides should now be bound to the columns.
80. Wash the columns 3× with 0.1% (v/v) aqueous TFA, centrifuge (3,000 g, 1 min, room temperature) and discard the flow-through.
81. Wash the columns 2× with 5% methanol (diluted in 0.1% aqueous TFA, 3,000 g, 1 min, room temperature) to remove excess TMTs.
82. Elute peptides from the desalting columns into fresh 2 mL LoBind® tubes by centrifugation (3,000 g, 1 min) using 50% ACN diluted in 0.1% aqueous TFA (2 × 300 μL).
83. Vacuum dry the desalted eluates (program “Aqueous” in the recommended evaporator).

**Pause point:** Desalted TMT-labeled peptides can be stored at −20°C for 6 months.

### Sample fractionation and LC-MS/MS analysis

**Timing: 2 days**

This section describes the fractionation of samples by high-pH reverse-phase high performance liquid chromatography (RP-HPLC). Fractionation enables less complex peptide samples to be prepared for subsequent analysis by LC-MS/MS.

84. Set-up of the Ultimate 3000 HPLC (Dionex) using the Chromeleon software. Buffers A and B should be less than 4-weeks old.
  a. Turn on UV lamp (wavelength 220 nm) at least 15 min prior to a run.
  b. Purge the system with Buffer A (10 mM ammonium formate, pH 9.5) and Buffer B (10 mM ammonium formate in 90% ACN, pH 9.5).
  c. Set the buffer composition to 98% A:2% B and the flow rate to 0.2 mL min^−1^ (these settings will be used in steps 85–87).
  d. Prime the syringe and wash the needle and fluidics using the commands in the Chromeleon software.
  e. Run the HPLC under these conditions for 15 min to fully equilibrate the system.
85. Run a standard containing caffeine and MRFA (Met-Arg-Phe-Ala peptide) to check the HPLC system is working correctly.
  a. Place collection tubes in the collection trays.
  b. Prepare the standard sample by diluting 2 μL of caffeine solution (1 mg/mL) and MRFA (1 mg/mL) in 100 μL buffer A in a HPLC sample tube. Place the sample tube in the samples tray.
  c. Run the sample using the following program:
    i. Load 25 μL sample in the column.
    ii. Elute peptides from the column with a gradient of 2–20% Buffer B over 8 min.
    iii. Over the following 37 min increase the % of Buffer B from 20%–47%.
    iv. Wash the column with 100% Buffer B for 15 min.
    v. Fractions (volume) should be collected from 1 – 80 min of the run.
  d. Discard the collected fractions from the standard run.
  e. Check the chromatogram for two peaks in minutes 12 and 14 that correspond to caffeine and MRFA, respectively.
86. Prepare and run the first sample.
  a. Resuspend the dry sample in 200 μL buffer A, vortex vigorously and then spin down at 20,000 g at 4°C for 15 min. Transfer 190 μL of supernatant to the sample tube and adjust to pH > 9 using aqueous ammonia (check pH with a pH indicator strip). Place the transfer tube in the sample tray.
  b. Label 80 collection tubes and add 20 μL 10% (v/v) aqueous FA to neutralize the pH of the fraction eluates. Place the collection tubes in the collection trays.
  c. Run the sample using the program detailed in 83c but loading 185 μL of sample.
  d. Combine the fractions from minutes 3–63 into 10 fractions by concatenating them. Discard fractions from minutes 1 and 2 as they usually contain TMT-related by products, salts and other contaminants and fractions from minute 64 onwards as they may contain detergent and further contaminants.
87. Repeat the run with a standard and continue running the following samples intercalating with standard runs.

***Note:*** Concatenate samples so that each contains eluted fractions collected at different points in the run. For example, fraction 3 would contain fractions eluted at 3, 13, 23, 33, 43, 53 and 63 min.

88. Vacuum dry the concatenated fractions (program “Aqueous” in the recommended evaporator).

**Pause point:** Concatenated fractions can be stored at −20°C for 6 months.

89. The mass spectrometer must be calibrated prior to analysis of samples.
90. Resuspend the concatenated samples in 50 μL 1% aqueous FA and then load 5 μL aliquots (at 15 μL min^−1^) onto the trap column previously equilibrated with 90% Buffer C (0.1% FA) and 10% Buffer D (0.1% FA in 90% ACN). Once samples are loaded, wash the column for 5 min with 5% Buffer D at 15 μL min^−1^.
91. Connect the trap column to the resolving column and elute peptides at 300 nL min^−1^ with a linear gradient (10% Buffer D at min 1, 18% at min 89, 27% at min 134 and up to 90% Buffer D at min 139). Wash the column with 90% Buffer D for 10 min and then re-equilibrate in 10% Buffer D for 25 min.
92. Use Orbitrap Eclipse in data-dependent mode using the following setup:
  a. MS1 scan cycles m/z range from 380–1,500 at resolution of 120,000 and standard automatic gain control (AGC).
  b. Followed by 15 sequential dependent MS2 scans with an isolation window set to 0.7 Da, maximum ion injection time at 50 ms and standard AGC.
  c. Then MS3 scans with a resolution of 50,000, an isolation window set to 0.7 Da, maximum injection time at 120 ms and 400% AGC target.
  d. Real-time search feature active using the *P. falciparum* 3D7 annotated proteins FASTA file (downloaded from PlasmoDB, latest version).

### Protein search, data analysis and hit selection

**Timing: 2 days**

This section describes the bioinformatic steps needed to find and quantify the proteins in the sample from the MS spectra using MaxQuant.^8^ This section also outlines the workflow to analyze the data, generate fold-change abundances for every protein, visualize these data, and select hits using the Perseus software.^8^

93. Place the RAW data files from MS analysis in a folder and open MaxQuant software (version 2.0.1.0).
94. Download the *P. falciparum* 3D7 annotated proteins FASTA file from PlasmoDB.org and the human proteome FASTA from Uniprot (accession 9606).

***Note:*** Other software can be used for this step (e.g., Thermo Proteome Discoverer).

***Optional:*** TMT labeling efficiency can be checked at this point using the parameters detailed below but by changing TMT reporters from variable to stable modification. This can be done in the “Configuration” tab in MaxQuant. The “Type” of experiment in the Group-specific parameters tab should also be set to MS1. TMT labeling efficiency can be performed on an aliquot of the sample prior to fractionation to ensure sufficient labeling before further analysis.

95. Use default settings except for the following:
  a. In the “Raw data” tab, RAW files are loaded and labeled according to their condition and replicate for example “experiment_1” using the “Set experiment” button. Fraction number can be selected for each of them using the “Set fraction” button and typing 1–10.
  b. In Group-specific parameters:
    i. Set Reporter ion MS3 under “Type” and choose the 10plex TMT option.
    ii. Change the correction factors in the table that will appear below according to the Reporter ion isotopic distributions table in the TMT10plex product data sheet.
    iii. Choose Oxidation (M), Acetyl (Protein N-term), Dioxidation (W), Deamidation (NW) and Gln->Pyro-Glu as variable modifications and Carbamidomethyl (C) as fixed modification under “Modifications”.
    iv. Select Trypsin/P enzyme under “Digestion”.
  c. In Global parameters:
    i. Add the FASTA files containing the protein sequences in the “Sequences” tab.
    ii. Set FTMS MS/MS Match tolerance to 10 ppm and ITMS MS/MS Match tolerance to 0.5 Da in the “MS/MS analyzer” tab.
    iii. Activate the “Match between runs” option in the “Identification” tab.
  d. Set the number of processors according to the capabilities of your system.

***Note:*** Analysis time is approximately 10 h using a 28-core computer.

96. The next steps are aimed at visualizing the results and selecting hits using the software Perseus.
  a. Open the Perseus software (version 1.6.15.0).
  b. Load the proteinGroups.txt file that can be found in the analysis folder\combined\txt folder using the “Generic matrix upload” button.
    ***Note:*** A screen with column names will appear on the left side.
  c. Select the “Reporter intensity corrected” columns 1–10 and add them to the “Main” box by clicking the “>” button. Click “Ok”. Troubleshooting 4.
97. The following screen is split into three panels: data on the left, matrix in the middle and details on the right. Every time a change is made to the data, a new matrix will appear in the matrix tree of the middle panel.
  a. Click on the “Filter rows” tab and select the option “Filter rows based on categorical columns”.
  b. Select “Only identified by site” and click on “OK”. This option will delete proteins identified only by modified peptides.
  c. Repeat this process selecting “Reverse” and then “Potential contaminant” to remove misidentified proteins and common contaminants from the protein list.
    ***Optional:*** If the data looks skewed after step 99 (the main cloud of unaffected proteins is not on the 0 line), normalization may be necessary before step 98 using the following step:
  d. In the “Normalization” tab select the option “Divide” and pick “columns” under the “Matrix access” tab and “Mean” under the “Divide by what” tab and click the “OK” button. Now the data values (TMT reporter intensities) will be normalized to the mean of each channel, removing variability due to differences in material.
98. To calculate the log2 fold-change DMSO vs drug-treated samples of every protein:
  a. Click on the “Basic” tab and select “Combine main columns”. A new menu will open.
  b. Write in the “Operation” section “log2(x/y)”.
  c. Select the “Reporter intensity” channels of the DMSO-treated sample and click on “>” to add them to the “x” box.
  d. Then select the “Reporter intensity” channels of the drug-treated samples and click on “>” to add them to the “y” box.
  e. Click “OK” and a new column will be created containing the log2 FC DMSO/Drug.
99. Under the “Visualization” tab, select “Scatter plot”, then select “Columns” in the “Matrix access” query.

***Note:*** A new tab labeled “Scatter plot” will appear beside the “Data” tab under the current matrix tab. Troubleshooting 5.

100. Open the “Scatter plot” tab, choose the “log2 fold-change” column in the “X” tab and the “Lin” option on the tab beside it.
  a. Then choose the “Intensity” column in the “Y” tab and select the “Log” option in the tab beside.
  b. The resulting graph will show the log2 fold-change values of all proteins along the X axis, sorted by log-scaled total protein intensity in the Y axis. As shown in expected outcomes, a main cloud of non-enriched proteins will populate the zero line on the X-axis. These proteins were bound to the beads but were affected by the presence of competitor in the treated sample.

***Note:*** Some proteins may be on the negative side of the graph, but these should be treated as noise.

101. Putative hits in the pulldown will typically have a log2 fold-change value greater than 2 in two independent experiments (i.e., low- and high-loading compound beads).

***Note:*** Depending on the noise level, this threshold can be increased, selecting only proteins that are clearly away from the main cloud of proteins.

**CRITICAL:** Proteins identified with less than 2 unique peptides can be removed from the analysis whereas proteins with 2 or 3 unique peptides should be regarded with caution. Typically, hits of a pulldown experiment are enriched and are identified with several peptides.

***Optional:*** Perseus has some statistical tools that can be used to find significantly enriched proteins as well, but we have not used them in our analysis. These analyses usually require at least two replicates.

***Alternatives:*** Other software can be used to visualize and identify significantly enriched proteins such as the Bioconductor R package DEqMS.

## Expected outcomes

After 24 h, high-loading drug beads should be loaded to a level equivalent to 60–80% of bead capacity (that is 60–80% of all NHS esters have reacted with the nucleophilic amine in the probe). The low-loading resin should be loaded to 5–15% of capacity, as determined by HPLC of the reaction supernatant after 24 h (steps 1–11). For example, DDD02355221-drug beads were prepared with 82% and 11% loading levels for the high- and low-loading drug beads respectively.^1^

Asexual blood stage parasites should reach the desired parasitemia of 8–12% approximately 2–4 days after transfer to HYPERflasks (steps 15–17). At that point, ring stage parasites will be present indicating a healthy culture with re-invasion of erythrocytes taking place.

After centrifugation of the parasite lysate (steps 28–31), there should be > 5 mg of protein in the supernatant.

After proteomic analysis of pulldown experiments (steps 43–101), a clear enrichment (typically log_2_ FC DMSO/Drug >2) of protein target(s) (red dot) should be apparent in the plot (Figure 6). Ideally, (for compounds with a single target) a single protein should be significantly enriched. However, in many if not most pulldown experiments several proteins will appear enriched. This may be explained by probe specifically binding to targets with differing affinity. Alternatively, the probe may specifically interact with a single target protein that interacts with, or is in complex with, several other proteins. For example, cyclins can be enriched by probes that bind specifically to cyclin-dependent kinases.^4^

Although pulldown experiments are robust and true targets are expected to be enriched, some false positive hits may appear enriched during the analysis. We recommend performing two independent experiments in order to remove the effect of noise. Some proteins may also consistently appear enriched using a given probe: these “sticky” proteins can be ruled out by performing a dose-response pulldown.

**Figure 5:**
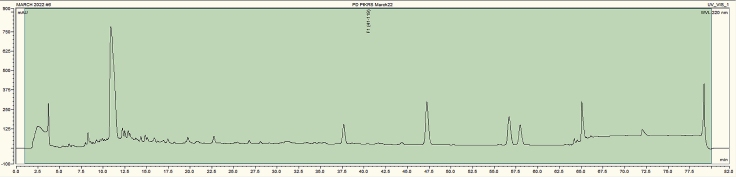
HPLC chromatogram generated using the Chromeleon software The chromatogram shows UV absorbance at 220 nm (expressed in milli-arbitrary units) over retention time (min).

The chromatogram produced in the HPLC analysis (step 86) is shown in Figure 5. This graph shows the UV intensity at 220 nm wavelength over time (80 min collection time). Peaks in min 1–2 correspond to salts and free TMTs and should be discarded. Peaks in minutes 64 and 65 should be discarded as they contain mostly undigested material and detergent. Peaks should be high and sharp, wider peaks may indicate contamination.

**Figure 6:**
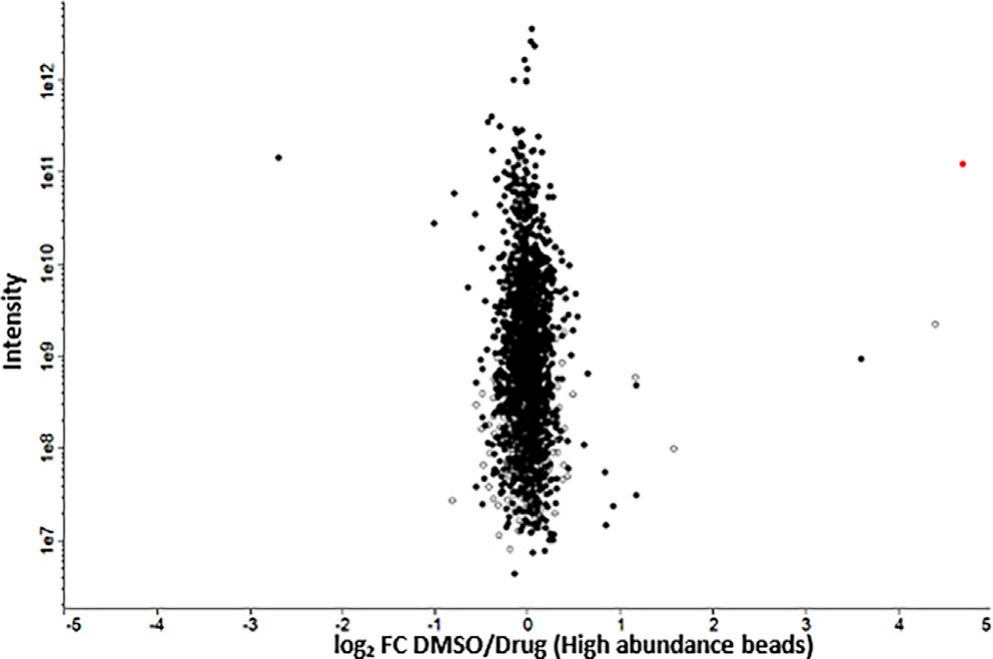
Graph representing data from a competitive pulldown experiment The graph, generated in Perseus, shows the log2 fold-change of proteins bound to DDD02355221-beads (high-loading level) incubated with *P. falciparum* lysate pre-treated with DMSO or competitor (DDD01510706) (Milne et al.^1^). Protein intensity is represented on the y-axis (log10 scale). Proteins identified by the presence of < 2 unique peptides are shown in white. The putative target of this probe, lysyl tRNA synthetase (KRS), is shown in red.

Figure 6 depicts representative data from a competitive pulldown graph generated with Perseus: a clear unaffected protein array is found along the x-axis 0 line (with a log_2_ FC DMSO/competitor close to 0), sorted by total intensity (y-axis, logarithmic scale). In this graph, the target has been labeled in red with all other proteins in black with the exception of proteins identified by the presence of <2 unique peptides which are shown in white. In this pulldown, 3 proteins have log_2_ FC value of >2, but two of these proteins were identified by the presence of 1 or 2 unique peptides and as a result were considered noise. Lysyl tRNA synthetase (KRS, red) was highly enriched and identified by the presence of more than 30 unique peptides.

## Limitations

The success of chemical pulldowns relies upon the affinity of the probe for its protein target(s). Therefore, probes with low binding affinities for their target proteins will not be significantly enriched from cell-free lysates.

For a pulldown to succeed, target protein(s) must be present in cell-free lysate in a conformation that enables probe binding and in sufficient quantity to be detected by MS. To maintain native conformation many proteins must remain part of a multi-protein complex or be embedded within a cell membrane. It should be noted that it is not always possible to maintain the integrity of all multi-protein complexes during lysate production. Membrane protein targets can also be lost in the preparation of standard lysates, however, if there is reason to believe that the target may be a membrane protein lysate preparation protocols can be tailored to enrich for these proteins specifically.

It is possible for probes to interact with, and pulldown, proteins that do not contribute to their mechanism of action thus complicating target deconvolution. This may be due to the inherent promiscuity of the probe leading to multiple interactions with off-targets or secondary targets. Performing pulldowns with varying concentrations of the competitor compound can help determine the relative affinity or K_d_ that the probe has for specific proteins. This information can be used to prioritize putative targets for further study.^4^

The significant chemistry resource and knowledge required to prepare appropriate probes for pulldown studies can be considered a limitation of this target identification approach since this resource is not available in all laboratories.

In some cases, it may be impossible to prepare probes for pulldown that retain the bioactivity and/or phenotype of the parent compound, i.e., to the SAR does not identify a solvent-exposed moiety in the compound of interest. In these instances, a label-free chemical proteomics method such as thermal proteome profiling might be more suitable.

The use of probes attached to a solid support is an inherent feature of chemical pulldowns. As such, increasing the concentration of probe in an experiment causes a concomitant increase in non-specific resin binding. This limitation is not shared by alternative chemical proteomics methods such as photoaffinity labeling.

This technique as described here is unable to identify non-protein targets.

## Troubleshooting

### Problem 1

Step 8. The standard curve is inaccurate (e.g., the equation derived from the standard curve is not calculating the correct concentration of the quality control samples, which are used to determine the accuracy of the standard curve).

### Potential solution

Remake and analyze the samples used to establish the standard curve, making sure to be precise (R^2^ > 0.95).

If the accuracy of the standard curve cannot be confirmed, then a qualitative assessment of compound attachment can be used, using the ratio of peak area at t = 0 and t = 24 h. In our experience, this qualitative assessment provides a good estimation of bead loading level.

### Problem 2

Step 11. No detectable decrease in the amount of probe in solution when attaching to the resin (compound is not attached).

### Potential solution

There are several potential causes of this issue.

**Figure 7:**
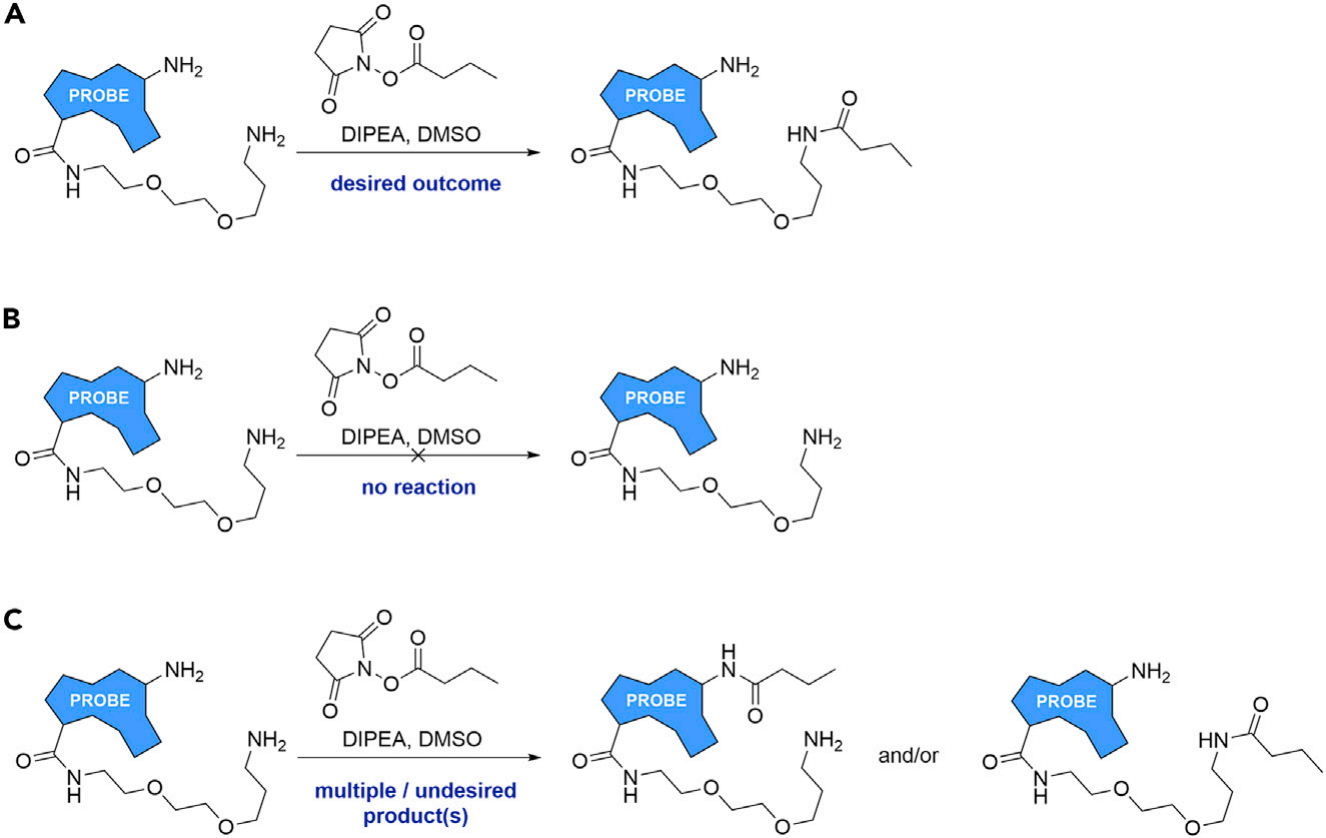
Potential outcomes of a model NHS- ester with an idealized pulldown probe (A) model ester reacts on the desired atom, the test reaction is successful and the drug bead-forming reaction should work. (B) The model reaction gives no product, the test reaction is unsuccessful and the drug bead-forming reaction is unlikely to work. (C) Reaction with a model ester gives multiple products, or an undesired product; the probe is poorly suited to forming a drug bead with NHS-activated Sepharose.

A solution-phase test reaction of the probe with a model NHS ester (e.g., succinimidyl butanoate) in DMSO may be conducted to check the amine of the PEG linker group is sufficiently reactive towards NHS-esters (Figure 7).^9^ If the model reaction gives no product (Figure 7B) then either change the chemistry used to attach the probe to the Sepharose resin (see below) or prepare an alternative probe.

If reaction with a model NHS-ester gives an undesired product, or a product mixture (Figure 7C) then consider preparing an alternative probe. Reaction with other nucleophilic groups will result in the probe being attached in an orientation that may not allow binding to its molecular target.

If model reactions demonstrate that the probe is reactive towards NHS esters, but the drug bead-forming reactions continue to be unsuccessful, the batch of resin may be expired/defective; purchase a new bottle of resin (ideally with a different batch/lot number).

If the problem persists, alternative attachment chemistry may be required, e.g., an azide-containing probe can be attached to a commercial alkyne-containing resin using a copper catalyzed click reaction.

### Problem 3

Step 23. Erythrocyte lysis is not complete leading to contamination of lysate with hemoglobin.

### Potential solution

When saponin completely lyses erythrocytes, the resulting pellet containing free parasites is characteristically dark brown in color. If partial lysis has occurred, some blood will be visible in the pellet giving a redder appearance. In this situation, additional saponin can be added to the pellet wash buffer during the first wash step. However, extra care should be taken not to incubate the parasites for too long with saponin, as this can lead to parasite membrane lysis.

### Problem 4

Step 96. Noisy pulldown.

### Potential solution

If Perseus analysis produces a very noisy graph (proteins around the log_2_ FC 0 axis are scattered beyond 2 and -2), check TMT labeling efficiency. If TMT labeling is fine, repeat the MS analysis loading more material if the normalized abundance is lower than 1 × 10^10^. If intensity is acceptable, repeat the pulldown with a different competitor concentration or resin loading level.

### Problem 5

Step 99. No proteins or very few proteins identified.

### Potential solution

If analysis identifies less than 500 proteins using our methodology, check TMT labeling efficiency. If TMT labeling efficiency is acceptable, repeat the MS analysis loading more material if the normalized abundance is lower than 1 × 10^10^. If this is fine, repeat the pulldown with a different competitor concentration or higher bead loading level, or using more cell lysate. We typically identify around 2000 proteins (85% *P. falciparum*, 15% Human proteins).

## Resource availability

### Lead contact

Further information and requests for resources and reagents should be directed to and will be fulfilled by the lead contact, Dr Susan Wyllie (s.wyllie@dundee.ac.uk).

### Materials availability

This study did not generate new unique reagents.

## Data Availability

Mass spectrometry raw files and their associated MaxQuant output files generated during this study are available at ProteomeXchange Consortium via the PRIDE partner repository (http://www.ebi.ac.uk/pride/archive/), under the identifier PXD033740 as listed in the key resources table.^10^

## Supplemental information

Data S1. Spreadsheet to calculate drug bead loading level This spreadsheet is used to calculate drug bead loading level from HPLC peak areas using a standard curve, see step 11.
